# Gratitude and health behaviours: A temporal affective self‐regulation resource analysis across 24 samples

**DOI:** 10.1111/bjhp.70103

**Published:** 2026-08-19

**Authors:** Fuschia M. Sirois, Jameson K. Hirsch, Alex M. Wood

**Affiliations:** ^1^ Department of Psychology Durham University Durham UK; ^2^ Department of Psychiatry and Behavioral Sciences, Quillen College of Medicine East Tennessee State University Johnson City TN USA; ^3^ School of Psychology and Therapeutic Studies Leeds Trinity University Leeds UK

**Keywords:** emotions, health‐related behaviours, motivation, psychological factors/processes

## Abstract

**Objectives:**

Although research increasingly highlights the relevance of gratitude for understanding consequential health outcomes, including health behaviours, the reasons why gratitude plays a role remain unclear. The current study investigated the extent to which gratitude is associated with the practice of health‐promoting behaviours, and the factors that contribute to this association. Guided by the Temporal Affective Self‐Regulation Resource model, we examined the contributions of positive and negative affect, and future time‐orientation (FTO) as self‐regulation resources for explaining the association between gratitude and health behaviours across multiple samples.

**Methods:**

Twenty‐four samples (*N* = 6967) from community, student and medical populations reporting correlations between state or trait gratitude and the practice of health‐promoting behaviours were included. Random effects meta‐analyses were conducted on the raw and semi‐partial correlations of gratitude with health behaviours controlling for positive and negative affect and FTO. Moderator analyses evaluated the impact of demographic, methodological and conceptual factors.

**Results:**

Gratitude was significantly associated with health‐promoting behaviours (*r*_avg_ = .270; 95% CIs [.24, .30]). Subgroup and meta‐regression moderator analyses were not significant. Meta‐analyses of the semi‐partial correlations revealed significant but lower magnitude associations between gratitude and health‐promoting behaviours after controlling for positive affect, negative affect and FTO.

**Conclusions:**

The current study provides initial evidence indicating that gratitude is associated with the practice of health‐promoting behaviours, and insights into the self‐regulation factors that contribute to this association. Taken together with previous intervention research, these findings highlight gratitude as an alternative, strengths‐based approach to health behaviour change across different populations and contexts.

**Statement of Contribution**

**What Is Already Known on this Subject?**

- Gratitude is linked to a number of consequential health outcomes including some health‐related behaviours.
- There is limited research that explains why and how gratitude may support health‐promoting behaviours.
- Although theory suggests that gratitude provides self‐regulation resources that can support health behaviours, this has not been tested.

**What Does this Study Add?**

- Gratitude is positively associated with different health behaviours across diverse samples.
- Higher positive affect and future orientation, and lower negative affect accounted for this link in part.
- Gratitude supports health behaviours through affective and temporal self‐regulation resources.

## INTRODUCTION

A growing evidence base has highlighted the importance of gratitude for a range of consequential health outcomes, including better health‐related quality of life (Toussaint et al., 2017), improved blood pressure, glycaemic control and asthma control (Boggiss et al., 2020), better sleep quality (Wood et al., 2009), as well as positive cardiovascular health outcomes, lower stress and lower inflammation (Cousin et al., 2021; Deichert & Fekete, 2025). Although the practice of positive health behaviours is one plausible route through which gratitude may positively impact health, the relevance of gratitude for health‐promoting behaviours is understudied (Sirois & Wood, 2025). This has led to an ambiguous evidence base (Hill et al., 2013; Legler et al., 2019; Millstein et al., 2016), which, although promising, does not provide clear or compelling insights into the question of how gratitude may promote a wide range of health‐related behaviours such as healthy eating and staying physically active.

Self‐regulation, a central process for initiating and maintaining health behaviours (Baumeister et al., 1994), may have relevance for understanding the role of gratitude in health‐promoting behaviours. For example, other positive psychological factors, such as self‐compassion, have been linked to health behaviours through self‐regulation resources, including high levels of positive affect and low levels of negative affect (Sirois et al., 2015). Future time perspective has also been identified as an important factor for supporting all stages of self‐regulation from goal setting to monitoring (Baird et al., 2021), and is therefore proposed to be a key self‐regulation resource for health behaviours alongside affective states (Sirois, 2015b). Emerging evidence indicates that gratitude is linked to each of these self‐regulation resources (Sirois & Wood, 2025). However, the immediate question of whether and why gratitude is linked to the practice of a range of key health behaviours, and if so, whether gratitude has incremental value above that accounted for by self‐regulation resources, remains to be addressed.

### Gratitude and health behaviours

Gratitude can be considered as a positive social emotion arising from the aid we receive from others (Wood et al., 2011) or as a life orientation (Wood et al., 2008). This latter conceptualisation views gratitude as a tendency ‘towards noticing and appreciating the positive in the world’ (Wood et al., 2010), which involves experiencing gratitude frequently, intensely and across a variety of situations (McCullough et al., 2002). That gratitude can be cultivated through engaging in reflective daily gratitude practices (e.g., Cheng et al., 2015) is particularly important when considering the potential of this positive quality for improving health‐related outcomes such as health behaviours.

Recent years have seen increasing interest in the role of gratitude for a range of consequential health outcomes. Although the evidence with respect to health behaviours is scant in comparison to that for other health outcomes, it nonetheless warrants further attention, especially as the samples and types of health behaviours investigated are limited. For example, in a large sample of adults, trait gratitude was associated with engaging in health‐promoting activities, such as healthy eating and exercise (Hill et al., 2013). Two longitudinal studies of patients who had experienced acute coronary syndrome (ACS) found similar results (Legler et al., 2019; Millstein et al., 2016), with dispositional gratitude at 2 weeks post ACS being associated with better medical adherence to health behaviour recommendations at 6 months. Notably, the findings remained significant after accounting for the effects of potentially confounding social and medical variables in one study (Millstein et al., 2016), but not the other (Legler et al., 2019).

Results are mixed, however, in the handful of studies that have examined the effectiveness of gratitude interventions for changing health‐promoting behaviours (see Boggiss et al., 2020 for a review). Gratitude writing interventions were effective for increasing healthy eating behaviour in high school students compared to a control group at the four‐week follow‐up (Fritz et al., 2019), and for reducing unhealthy eating behaviours in female undergraduate students compared to the cognitive restructuring intervention and the control group (Wolfe & Patterson, 2017). However, in an 8‐week randomized controlled trial of a gratitude intervention targeting self‐care behaviours and glycaemic control in adolescents with Type 1 diabetes, self‐care did not improve for those in the intervention group compared to the control group (Schache et al., 2020). Glycaemic control, which relies mainly upon dietary behaviours, did show small but significant improvements for the intervention group, providing some support for the potential role of gratitude in promoting healthy eating.

### Gratitude and the self‐regulation of health behaviours

An important yet understudied area within the gratitude and health literature is why gratitude might play an important role in health behaviours. Building on the model of positive psychological well‐being (Boehm & Kubzansky, 2012), Schache et al. (2019) proposed positive affect as a key mechanism through which gratitude promotes engagement with health behaviours. Synergies among gratitude, positive affect and social support were posited to directly influence restorative processes that included health behaviours. However, this proposed framework has yet to be formally tested, nor does it consider other factors known to influence health behaviours.

Defined as the regulation of behaviours, thoughts and emotions directed towards reaching goals (Carver & Scheier, 1982), self‐regulation is a key process underlying the practice of health behaviours that may have relevance for understanding how gratitude can support health‐promoting behaviours. Goal setting and progress monitoring are two core processes within self‐regulation that can be facilitated or hindered by affective states and temporal thinking (Baird et al., 2021; Tice & Bratslavsky, 2000). With respect to health behaviours, goal setting requires setting long‐term health goals and considering the future consequences of one's current actions (Dassen et al., 2016; Joireman et al., 2012), whereas progress monitoring can activate positive emotions which can sustain motivation (Løvoll et al., 2017). In this respect, future‐oriented thinking and positive affect can be considered self‐regulation resources that support health behaviour change (Sirois, 2015a, 2015b). Equally important is managing the negative affective states such as frustration, stress or anxiety that can arise after lapses and disrupt self‐regulation by shifting focus away from the long‐term goal of achieving health goals to the short‐term goal of reducing negative states, which can derail goal persistence and encourage procrastination of health goals (Sirois & Pychyl, 2013). Thus, low levels of negative affect are also important for supporting successful behaviour change.

Evidence points to clear links between gratitude and positive and negative affect, as well as future‐oriented thinking. Gratitude interventions have been shown to be effective for increasing levels of positive affect in a clinical population (Krentzman et al., 2015), and for reducing negative states (Krentzman et al., 2015; Portocarrero et al., 2020). Similarly, dispositional gratitude is linked to lower levels of depression, anxiety and stress in both general and medical populations, in cross‐sectional and longitudinal studies (Cregg & Cheavens, 2021; Sirois & Wood, 2017). Findings from correlational and longitudinal research converge with those from neuropsychological studies to suggest that gratitude is reliably associated with future‐oriented thinking. Correlational studies demonstrate that gratitude is associated with higher levels of future‐oriented thinking (Allemand & Hill, 2016; Casu et al., 2020; Przepiorka & Sobol‐Kwapinska, 2021; Szcześniak & Timoszyk‐Tomczak, 2018) and similarly, in a two‐week prospective study, average daily state gratitude was significantly associated with higher average daily future‐time perspective (Allemand & Hill, 2019). Evidence from neuropsychological studies indicates that both dispositional gratitude and gratitude interventions are associated with lasting and measurable changes in activity in functional brain regions implicated in prospective thinking and predicting the outcomes of one's actions (Fox et al., 2015; Kini et al., 2016).

**FIGURE 1:**
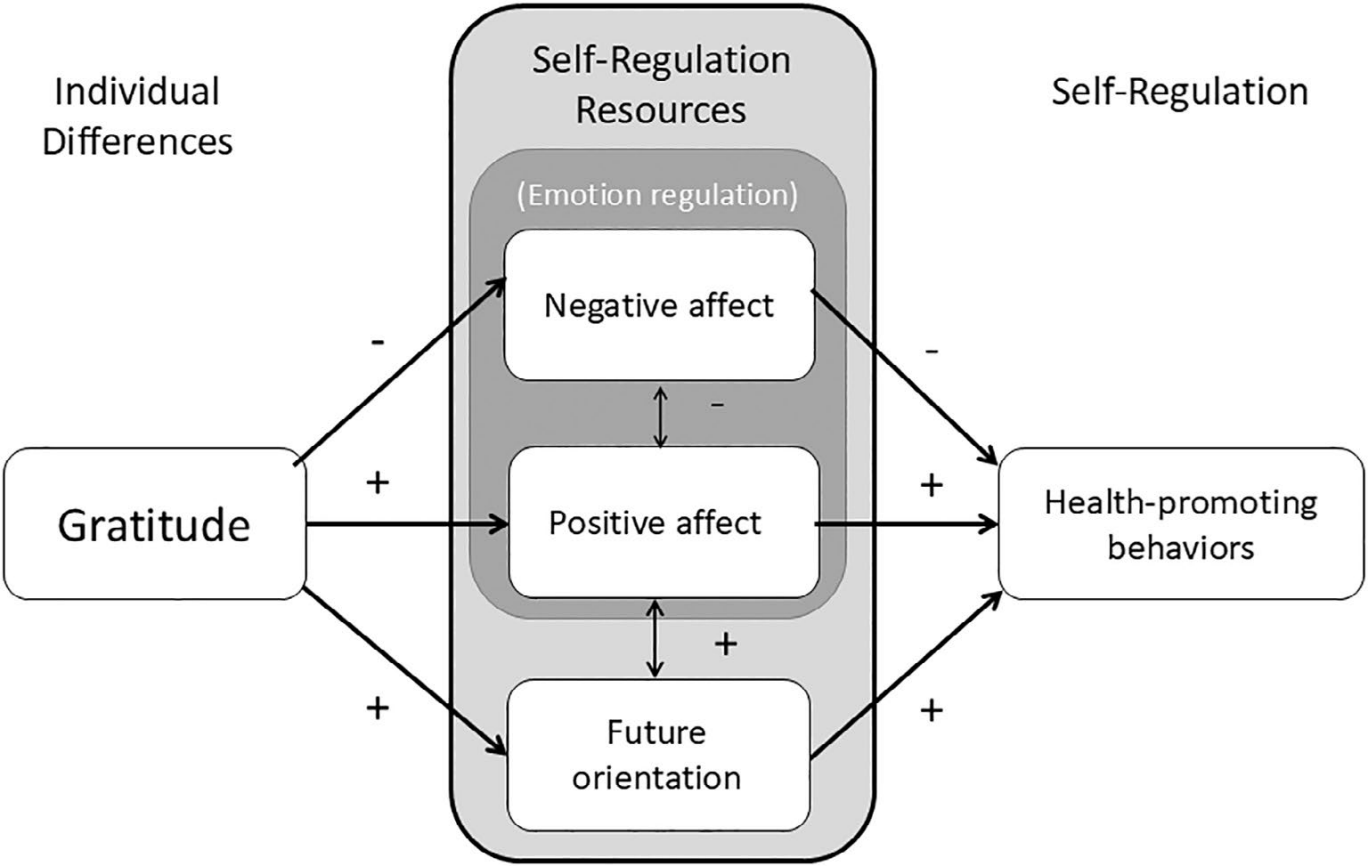
The temporal affective self‐regulation resource model (TASRR) as applied to understanding gratitude and health behaviours. Reprinted with permission from Sirois & Wood (2025).

Given the above evidence, the Temporal Affective Self‐Regulation Resource model (TASRR; Sirois, 2015b, 2016) is one conceptual framework that is appropriate for explaining why gratitude is linked to health behaviours (Sirois & Wood, 2025). The TASRR outlines how temporal thinking and affective states associated with personality traits and qualities can support or hinder the self‐regulation of health behaviours (see Figure 1). Crucially, the TASRR acknowledges the synergistic, dynamic and mutually reinforcing links between affective states and temporal thinking, viewing these as potential resources to facilitate the successful self‐regulation of health behaviours. For example, theory positions positive affect as an important internal resource for down‐regulating negative mood and supporting self‐regulation (Fredrickson, 2001), and evidence suggests that positive affect can enhance future‐oriented thinking and vice versa, via promoting abstract thinking which, in turn, supports positive affective states (Critcher & Ferguson, 2011; Fredrickson, 2001; Rösch et al., 2021; Sirois et al., 2015). These synergies are juxtaposed with those of negative affect, which foreshortens the action‐contingency horizon and creates a temporal short‐sightedness or myopia that focuses attention on immediate threats and concerns at the expense of longer range goals (Sirois, 2014), including health behaviours (Sirois, 2015b). According to the TASRR model, the associations of gratitude with both positive affect and future‐oriented thinking, and to low levels of negative affect, explain why gratitude can facilitate health‐promoting behaviours.

### The current study

Theory and evidence suggest that gratitude may facilitate the practice of health behaviours through links to future‐oriented thinking and positive affect, and low levels of goal‐derailing negative affective states. Accordingly, the current study investigated the extent to which trait and state gratitude are associated with the practice of health‐promoting behaviours, and the contribution of temporal and affective self‐regulation resources in this association. Consistent with the TASRR model, we examined the contribution of positive and negative affect and future orientation for explaining the association between gratitude and health behaviours across previously collected data sets by examining their semi‐partial correlations. Specifically, we investigated the extent to which gratitude had shared variance with health behaviours after partialling out the variance of each of the self‐regulation resource variables from gratitude, with the expectation that removal of the contribution of these variables would attenuate the magnitude of the association between gratitude and health behaviours.

Unlike traditional meta‐analyses which systematically review the extant research literature and then quantify the effects across studies, we used an approach we refer to as Meta‐analysis of [one's] Own Data or ‘MOD’ which uses the statistical technique of meta‐analysing data across data sets to estimate the average effects. In this respect our use of the term ‘meta‐analysis’ is closer to Glass's (1977) conception of this technique as being the statistical analysis of the results from a series of studies. The MOD approach involved including unpublished data sets and data from previously published papers that did not report the variables of interest from the researchers' labs. The effects were then extracted and moderator information recorded in the same way as one would for a traditional meta‐analysis. Although analysing data from one's own labs raises the issue of whether there are lab‐specific biases influencing the findings, previous research using a mixed MOD approach (i.e., conducting a traditional meta‐analysis of published literature alongside a meta‐analysis of lab data) found that there were no significant differences in the effects garnered from a systematic search versus those from the authors' labs (Sirois et al., 2017).

One advantage of the MOD approach is that it provides an opportunity for a robust test of not only the bivariate association between the variables of interest, but also of the associations after partialling out the contributions of other variables to better understand the unique contributions of key variables (see Sirois et al. (2015) for an example). This approach is also advantageous when the published literature is limited with respect to the variables of interest and would therefore not be conducive to taking a traditional meta‐analytic approach. We then statistically meta‐analysed these associations to quantitatively summarize their magnitude and significance, and examined whether the associations varied as a function of demographic (age and percent female), methodological (sample type) and conceptual (trait vs. state) moderators. In the absence of strong theory or research to suggest otherwise, we expected that each of these moderator tests would be non‐significant.

## METHODS

The present research analysed data from 24 independent samples (total *N* = 6967), comprised of 6 undergraduate and graduate student, 13 community adult and 5 medical/chronic illness (S6, S24: fibromyalgia; S7: chronic fatigue syndrome; S12: cancer; S14: American war veterans) samples. Data were collected over a nine‐year period from 2011 to 2020 as part of a larger research program focused on the correlates of gratitude and health. Ethical clearance for the data collection was obtained through the three respective Institutional Research Ethics Boards (US, Canada and the United Kingdom). Analysis of the data was pre‐registered on the Open Science Framework (OSF) (10.17605/OSF.IO/A5QFV).

All samples completed online surveys. The 13 community‐dwelling adult samples were recruited from online and community sources, and the undergraduate and graduate student samples were recruited from two different post‐secondary institutions. Samples were recruited using a variety of similar means including adverts posted on University volunteer lists, on online psychology research websites, and on social media. Recruitment for the five chronic illness samples additionally utilized notices placed on relevant online support boards, and in the UK Fibromyalgia newsletter (S6). All participants gave consent prior to participating and were given a chance to win gift cards of varying values as a participation incentive or student participation credits for student samples.

Table 1 summarizes the demographic characteristics of each of the 24 samples. The samples were predominantly white and female.

**TABLE 1:** Demographic characteristics of the 24 independent samples (*N* = 6967).

Sample	*N*	Percent female	Percent White	Age (years)		Education level (%)		
				*M*	*SD*	High school	College/university	Graduate school
1	548	45.6	79.4	36.84	11.80	13.5	71.3	15.2
2	130	52.3	–	43.37	12.67	21.5	59.8	18.7
3	172	82.0	82.6	31.08	12.68	13.4	49.4	37.2
4	177	74.0	95.6	33.81	17.63	12.4	79.7	7.9
5	161	76.5	80.9	30.26	11.72	9.3	67.9	22.8
6	165	52.8	86.5	42.26	13.96	17.7	63.4	18.9
7	84	85.7	94.9	35.40	14.78	11.9	60.7	27.4
8	396	86.1	74.7	21.16	4.07	0.0	100.0	0.0
9	289	70.9	91.6	21.08	4.41	0.0	100.0	0.0
10	238	65.3	85.2	21.41	5.12	9.6	90.3	0.0
11	75	81.6	77.6	22.42	4.85	11.8	77.6	10.5
12	176	61.7	92.6	59.61	12.28	5.1	65.7	29.1
13	644	69.3	75.5	30.62	12.31	8.2	51.6	40.1
14	456	30.1	89.0	51.37	16.59	23.1	48.1	28.8
15	548	51.4	81.0	36.37	11.68	12.2	73.5	14.3
16	412	78.4	84.3	28.99	10.86	7.8	55.1	37.1
17	163	67.5	89.6	37.93	13.02	6.2	46.9	46.9
18	234	74.1	74.1	25.96	11.45	19.7	62.2	18.0
19	220	74.5	–	29.60	10.95	–	–	–
20	706	69.2	79.2	20.70	4.96	–	–	–
21	353	47.1	76.2	35.84	11.25	27.9	65.0	7.1
22	134	68.7	–	–	–	–	–	–
23	307	35.8	57.3	30.78	17.4	10.1	56.2	33.7
24	179	41.2	87.7	42.01	14.0	17.4	63.5	19.1

### Measures

Participants completed standard demographic questions about age, gender, ethnicity and education level. The means, standard deviations and Cronbach alphas for all the scales are presented in Table 2.

**TABLE 2:** Summary of the characteristics of the study variables for the 24 independent samples (*N* = 6967).

Sample (*N*)	Gratitude			Health behaviours			Positive affect			Negative affect			Future orientation		
	M	(*SD*)	α	M	(*SD*)	α	M	(*SD*)	α	M	(*SD*)	α	M	(*SD*)	α
S1 (548)	5.43	1.19	.89	3.39	.68	.73	–	–	–	–	–	–	3.73	0.87	.82
S2 (170)	5.34	1.11	.87	3.29	.68	.69	–	–	–	–	–	–	–	–	–
S3 (172)	5.89	0.90	.82	3.53	.57	.65	–	–	–	–	–	–	–	–	–
S4 (177)	3.35	1.29	.92	3.84	.57	.71	3.04	.91	.92	1.86	.78	.90	3.46	.68	.81
S5 (161)	3.61	1.01	.91	3.46	.5	.57	3.37	.84	.91	1.93	.75	.93	–	–	–
S6 (165)	5.51	1.09	.84	3.26	.71	.71	–	–	–	–	–	–	–	–	–
S7 (84)	5.44	1.07	.85	3.42	.65	.67	–	–	–	–	–	–	–	–	–
S8 (396)	3.26	0.87	.90	3.55	.60	.71	3.23	.67	.87	2.40	.70	.87	–	–	–
S9 (289)	3.38	1.08	.91	3.78	.52	.67	3.14	.84	.90	2.02	.71	.88	–	–	–
S10 (238)	5.75	1.15	.91	3.12	.63	.74	30.94	22.6	.79	59.50	22.4	.91	3.31	.55	.78
S11 (75)	5.97	1.02	.87	3.57	.70	.74	–	–	–	–	–	–	3.52	.63	.81
S12 (176)	6.34	.78	.85	3.59	.62	.72	–	–	–	–	–	–	–	–	–
S13 (644)	5.67	1.00	.83	3.73	.53	.66	4.68	1.47	.80	2.87	1.48	.87	3.40	.70	.88
S14 (456)	8.01	1.94	–	3.10	.75	.77	–	–	–	–	–	–	3.63	.93	.74
S15 (548)	7.45	2.16	–	3.67	.65	.71	56.23	23.9	.85	19.28	18.9	.90	3.55	.77	.92
S16 (412)	5.70	.94	.81	3.78	.51	.65	3.64	1.02	.76	2.42	1.03	.85	2.79	1.04	–
S17 (163)	5.56	.98	.84	3.58	.55	.65	3.44	1.02	.79	2.36	1.00	.86	5.07	1.67	–
S18 (234)	5.66	1.04	.80	3.51	.66	.72	2.61	.91	.88	1.68	.73	.90	4.49	1.70	–
S19 (220)	5.39	1.13	.82	3.50	.57	.64	2.41	.94	.84	1.96	.98	.90	4.51	1.72	–
S20 (706)	5.66	1.13	.84	3.21	.62	.68	–	–	–	–	–	–	3.62	.59	.81
S21 (353)	5.19	1.36	.90	3.42	.70	.76	–	–	–	–	–	–	4.03	.78	.78
S22 (134)	5.90	.80	.80	3.64	.50	.66	–	–	–	–	–	–	–	–	–
S23 (307)	5.01	1.03	.75	3.47	.60	.68	–	–	–	–	–	–	–	–	–
S24 (179)	5.40	1.18	.84	3.24	.72	.72	–	–	–	–	–	–	–	–	–

*Note*: For samples 4, 5, 8 and 9, the original 10‐item PANAS positive and negative affect scales embedded within the PANAS‐X assessed affect. Samples 10 and 15 used a 10‐item Visual Analogue version of the Positive and Negative Affect Schedule (PANAS; Watson et al., 1988); for the remaining samples the 10‐item PANAS positive and negative affect subscales (5‐point scale) assessed affect.

### Gratitude

Eighteen samples (Samples 1–3, 6, 7, 10–13, 16–24) completed the 6‐item Gratitude Questionnaire (McCullough et al., 2002), that assesses gratitude as a life orientation towards noticing and appreciating the positive in life. Items (two reverse coded) are rated on a 1 (strongly disagree) to 7 (strongly agree) scale. Items such as ‘I feel thankful for what I have received in life,’ and ‘Long amounts of time can go by before I feel grateful to something or someone’ assess how frequently and intensely participants experience gratitude. The GQ‐6 has demonstrated good internal consistency previously (McCullough et al., 2002). Two samples (14 and 15) completed a single item measure of dispositional gratitude with the question ‘I am a grateful person’ rated on a 10‐point scale ranging from 1 = ‘not a all’ to 10 = ‘very much.’ Four samples (4, 5, 8 and 9) completed three items from the PANAS‐X gratitude adjective scale that were embedded within the expanded positive and negative affect measure (see below) (Watson et al., 1988).

### Health behaviours

The frequency of practicing health‐promoting behaviours was assessed in all 24 samples using the 12‐item Wellness Behaviours Inventory (WBI; Sirois, 2025). Items such as ‘I eat fresh fruits and/or vegetables’ and ‘I walk as much as possible, for example, I take the stairs not the lift, etc.’ are scored on a 5‐point scale with responses ranging from 1 (less than once a week or never) to 5 (every day of the week). Two items (3 and 10) are reverse scored before calculating the mean. The WBI general wellness behaviours mean is based on 10 of the 12 items, and excludes two items related to vitamin and supplement use (items 9 and 12). After reverse scoring 2 items, the 10 items are averaged to create an overall index of how frequently people engage in health‐promoting behaviours organized across four conceptual categories: healthy eating, regular exercise, sleep behaviour and stress management. The WBI has demonstrated good construct validity in previous studies, positively correlating with other preventive health behaviours, heath behaviour intentions, physical health and perceived control over health (Dunne et al., 2018; Sirois, 2007; Sirois et al., 2003, 2015; Sirois & Hirsch, 2015). Internal consistency for the WBI is also adequate, ranging from .64 to .75 in previous research (Sirois, 2025).

### Positive and negative affect

Eleven of the 24 samples completed one of four versions of the Positive and Negative Affect Schedule (Watson et al., 1988) to assess state negative and positive affect. Sample 18 completed the original 20‐item PANAS (Watson et al., 1988), which consists of 20 mood adjectives, 10 of which assess state positive affect and 10 that assess state negative affect. Participants rate their current mood on a 5‐point Likert scale ranging from 1 (*very slightly or not at all*) to 5 (*extremely*). The 20‐item PANAS has demonstrated good discriminative and internal reliability (alpha = .88).

Samples 10, 13, 15, 16, 17 and 19 completed a 10‐item abbreviated version of the PANAS (Watson et al., 1988), with 5 items for positive affect and 5 items for negative affect. However, Samples 10 and 15 rated the 10 PANAS items on a visual analog scale from 0 to 100, whilst the remaining samples rated items using the original 5‐point Likert scale.

Samples 4, 5, 8 and 9 completed the expanded 36‐item PANAS X scale (Watson & Clark, 1994), which included the original PANAS items plus additional positive and negative affect adjectives. For consistency, only the items from the original 10‐item negative affect and 10‐item positive affect scales were used to calculate state negative and positive affect scores in these samples.

### Future time orientation

Of the 13 samples that completed a measure of future time orientation, four (S1, S4, S20 and S21) completed the short 22‐item version of the Zimbardo Time Perspective Inventory (ZTPI‐S; D'Alessio et al., 2003), 2 (S11 and S15) completed the 12‐item Consideration of Future consequences scale (CFC; Strathman et al., 1994), 4 (S16 – S19) completed the single‐item Future Self‐Continuity Scale (FSC; Ersner‐Hershfield et al., 2009) and the remaining three samples (S10, S13 and S14) completed two of the above measures.

The 9‐item future subscale of the ZTPI includes items such as ‘I believe that a person's day should be planned ahead each morning’, which are rated on a 5‐point scale ranging from 1 (very uncharacteristic of me) to 5 (very characteristic of me). The future subscale had demonstrated adequate internal consistency in previous research, Cronbach alphas from .85–.94 (Sirois, 2014). The CFC (Strathman et al., 1994) is a unidimensional scale that assesses individual differences in the extent to which immediate versus distant consequences of behaviour are considered. High scores on the CFC indicate higher future time orientation as illustrated via statements such as ‘I consider how things might be in the future, and try to influence those things with my day‐to‐day behavior’ and ‘I am willing to sacrifice my immediate happiness or well‐being in order to achieve future outcomes’. Items are scored on a 5‐point Likert‐type scale, with response options ranging from 1 (extremely uncharacteristic) to 5 (extremely characteristic). The CFC scale has demonstrated good internal consistency in previous studies with Cronbach alphas ranging from .80 to .86 (Strathman et al., 1994). The FSC (Ersner‐Hershfield et al., 2009) asks participants to rate how connected and close they feel to a future self at a given time point in the future on a 7‐point scale that consists of two circles at each point that range from depicting no overlap between the current and future self, to depicting almost complete overlap. The time frame for the samples that completed this measure was set at 6 months in the future.

### Analytic strategy

Data was first analysed using SPSS version 28. Pearson's correlation was calculated for the associations of gratitude with health behaviours (*r*_*y1*_), and each of the self‐regulation resource factors (positive affect, negative affect and future time‐orientation (FTO); *r*_*1.2*_), and for the association of health behaviours with each of the self‐regulation resource factors (*r*_*y2*_) in each sample that measured these variables. Next, we calculated the semi‐partial correlations of gratitude with health behaviours adjusting individually for the contribution of each of the self‐regulation resource factors (positive affect, negative affect and FTO).

$${\mathrm{Sr}}_{1}=\frac{r_{y1}-r_{y2}*r_{12}}{\sqrt{1-r_{12}^{2}}}$$

This provided an estimate of the unique contribution of gratitude to health behaviours after accounting for the shared variance between gratitude and each of these factors and yielded six sets of adjusted effects to meta‐analyse. Semi‐partial correlations were only calculated for samples that used a trait measure of gratitude to avoid inflation of effects due to measurement bias (Baumgartner et al., 2021), where both gratitude and positive/negative affect were measured with the same affect measure (i.e., the PANAS). The magnitudes of the effect sizes were evaluated using Cohen's (1988) standards, with *r* = .10 considered a small sized effect, *r* = .30 considered a medium sized effect, and *r* = .50 considered a large sized effect.

We took a multi‐step approach to examine the associations of gratitude with health behaviours. The average association between gratitude and health behaviours across the 24 samples was estimated using a random effects model meta‐analysis conducted with Comprehensive Meta‐analysis (CMA), Version 2 software, which transforms the individual correlation coefficients into Fisher's *z* scores prior to the effects being meta‐analysed.

To assess any potential measurement bias in the results for the semi‐partialled effects obtained from analysing a subset of the samples, a moderator analysis was also planned to compare the unadjusted raw correlations among the samples that did and did not include a measure of positive and negative affect or future time orientation.

The variability in effect sizes between samples was evaluated with two approaches to determine whether the planned subgroup moderator analyses were warranted. First, the heterogeneity statistic, *Q*, assessed the degree of variability among the pool of effect sizes. Moderator analysis is warranted if this statistic is associated with a large confidence interval. Second, the *I* ^*2*^ statistic was used to estimate the proportion of variability present that is not due to sampling error within studies. As a general rule, *I* ^*2*^ values of 25 percent reflect low heterogeneity, 50 percent reflect moderate heterogeneity, and 75 percent or more reflect high heterogeneity.

Moderator analyses were planned to test the role of demographic (age and percent female), methodological (sample type), and conceptual (trait vs. state) moderators on the associations and semi‐partial effects for gratitude and health behaviours. These analyses were only conducted if subgroups included three or more studies in line with Card's (2012) caution regarding the reduction of statistical power and difficulties in detecting meaningful group differences when there are too few studies in a subgroup. Moderator analyses were conducted with a mixed effects approach where the combined subgroups were first analysed with a random effects model to further assess heterogeneity within each subgroup, and then combined using a fixed effects model to assess the heterogeneity between subgroups. A mixed effects meta‐regression (method of moments) analysis was used to assess the potential moderating effects of age and gender, as age was recorded as a continuous variable, and sex recorded as the percentage of the sample that was female. Meta‐regressions were only conducted if 10 or more studies were included in the pool of studies to be analysed.

To estimate the number of studies with null results that would have to be included in the meta‐analysis to render the current findings non‐significant, a Failsafe *N* was calculated. Although all of the data sets meta‐analysed in the current study were unpublished, it was still important to calculate the Failsafe *N* because there were a relatively small number of samples included in the overall analysis, and because other researchers were not contacted to obtain other unpublished studies. Rosenthal's (1979) guidelines were followed for determining an adequately high fail‐safe *N*. Accordingly, the Fail‐safe N should be greater than 5 *k* + 10, where *k* = the number of studies included.

## RESULTS

The results for the meta‐analysed correlations and semi‐partial correlations for the association of gratitude with health behaviours, and the meta‐analysed associations of gratitude with positive and negative affect and future time orientation, are presented in Table 3.

**TABLE 3:** Meta‐analysed effect sizes among gratitude (GR) and health behaviours (WBI) controlling for positive affect, negative affect (NA), and future time orientation across 24 independent samples (total *N* = 6967).

Sample	*N*	Gratitude measure	FTO measure	GR‐WBI	GR‐FTO	GR‐PA	GR‐NA	GR‐WBI	GR‐WBI	GR‐WBI
				*r*	*r*	*r*	*r*	*Sr*_*PA*_	*sr*_*NA*_	*sr*_*FTP*_
1. Community	548	GQ6	ZTPI/FSC	.351	.435	**–**	**–**	–	–	.214
2. Community	130	GQ6	**–**	.469	–	–	–	–	–	–
3. Community	172	GQ6	–	.285	–	–	–	–	–	–
4. Community	177	PANAS	ZTPI	.325	.260	.739	−.028	–	–	.176
5. Community	161	PANAS	–	.362	–	.724	−.254	–	–	–
6. Fibromyalgia	165	GQ6	**–**	.208	–	–	–	–	–	–
7. CFS	84	GQ6	**–**	.285	–	–	–	–	–	–
8. Student	396	PANAS	–	.285	–	.729	−.151	–	–	–
9. Student	289	PANAS	**–**	.118	–	.695	.048	–	–	–
10. Student	238	GQ6	CFC	.202	.264	.338	−.310	.117	.144	.157
11. Student	75	GQ6	CFC	.396	.168	–	–	–	–	.341
12. Cancer	176	GQ6	**–**	.088	**–**	**–**	**–**	–	–	–
13. Community	644	GQ6	CFC/FSC	.302	.287	.516	−.391	.121	.175	.218
14. Veterans	456	Single item	ZTPI/FSC	.270	.296	**–**	**–**	–	–	.182
15. Community	548	Single item	ZTPI/FSC	.184	.300	.505	−.466	.073	.051	.112
16. Community	412	GQ6	FSC/FS closeness	.340	.109	.423	−.287	.201	.236	.334
17. Community	163	GQ6	FSC/FS closeness	.185	.157	.549	−.217	.088	.159	.324
18. Community	234	GQ6	FSC/FS closeness	.238	.307	.326	−.298	.161	.169	.172
19. Students	220	GQ6	FSC	.362	.172	.421	−.222	.224	.311	.340
20. Students	706	GQ6	ZTPI	.229	.448	–	–	–	–	.147
21. Community	353	GQ6	ZTPI‐S	.246	.438	–	–	–	–	.125
22. Community	134	GQ6	–	.374	–	–	–	–	–	–
23. Community	307	GQ6	–	.242	–	–	–	–	–	–
24. Fibromyalgia	179	GQ6	–	.237	–	–	–	–	–	–
Meta‐analysis results										
Average *r* (*k*)				.270	.292	.451	−.251	.135	−.174	.208
95% CI				[.24, .30]	[.23, .36]	[.32, .56]	[−.34, −.16]	[0.09, .18]	[.11, .24]	[.17, .25]
*N*				6967	4774	3620	3620	2459	2459	4774

*Note*: CFC = Consideration of Future Consequences (Strathman et al., 1994); FSC = Future Self continuity (Ersner‐Hershfield et al., 2009); GQ6 – Gratitude scale (McCullough et al., 2002); PANAS – Gratitude items from the Positive Negative Affect Schedule (Watson et al., 1988; Watson & Clark, 1994); ZTPI = Zimbardo Time Perspective Inventory, future subscale (Zimbardo & Boyd, 1999); ZTP‐S – Zimbardo Time Perspective Inventory, short, future subscale (Zhang et al., 2013).

### Gratitude and health Behaviours

The overall meta‐analysis of the effects for the 24 samples (total *N* = 6967) revealed a significant and positive small to moderate‐sized average association between gratitude and the frequency of practicing health behaviours (see Table 3). The test of heterogeneity revealed there was a significant amount of unexplained variability among the unadjusted effect sizes, *Q* (23) = 45.71, *p* < .01; *I* ^*2*^ = 49.69%, indicating that moderator analyses were warranted.

### Gratitude, positive affect and health Behaviours

The meta‐analysis of the 11 samples (total *n* = 3620) that included a measure of state positive affect revealed the expected positive association between gratitude and positive affect, with a high degree of variability among the effects, *Q* (10) = 209.57, *p* < .0001; *I* ^*2*^ = 95.23%. For the 7 samples which included only measures of trait gratitude, the meta‐analysis of the semi‐partial correlations between gratitude and health behaviours, accounting for the contribution of positive affect, found that gratitude remained on average positively and significantly associated with health behaviours, with the magnitude of the average adjusted effect reduced to a small effect compared to the small to moderate sized effect for the unadjusted average effect. The tests of heterogeneity revealed a non‐significant and low degree of variability among the effects, *Q* (6) = 6.60, *p* = .36; *I* ^*2*^ = 9.12%, indicating that moderation tests were not necessary.

### Gratitude, negative affect and health Behaviours

Across the 11 samples that included a measure of negative affect, the analysis revealed the expected significant and negative associations between gratitude and negative affect, with a significant degree of heterogeneity among the effects *Q* (10) = 73.11, *p* < .0001; *I* ^*2*^ = 86.32%. The meta‐analysis of the semi‐partial associations of trait gratitude and health behaviours, adjusted for negative affect, revealed that gratitude remained on average positively and significantly associated with health behaviours with a small‐sized average effect. Similar to the adjusted effects for positive affect, the magnitude of the average adjusted effect was noticeably reduced compared to the unadjusted average effect. The tests of heterogeneity were significant, *Q* (6) = 14.91, *p* < .02; *I* ^*2*^ = 59.78%. However, given the small pool of samples for this analysis, moderator tests were not conducted.

### Gratitude, future time orientation and health Behaviours

The meta‐analysis of the 13 samples which included a measure of future time orientation revealed a small to moderate and significant positive association with gratitude, with a significant, high degree of heterogeneity, *Q* (12) = 67.83, *p* < .001; *I* ^*2*^ = 82.31. The analysis of the semi‐partial correlations of gratitude with health behaviours accounting for the contribution of future time orientation found a significant, positive average association that was attenuated in magnitude from the unadjusted average effect size. There was also a moderate and significant degree of variability among the effects, *Q* (12) = 27.93, *p <* .01; *I* ^*2*^ = 57.04%, suggesting moderator analyses were warranted. However, as the minimum threshold of three studies per subgroup for moderator analysis was not met, no moderator tests for sample type or FTO measure used were conducted.

### Moderator analyses – Included versus excluded studies

The first moderator analyses assessed whether the magnitude of the unadjusted effects among the samples that tested the effects adjusted for positive and negative affect and for future time orientation varied significantly from those that were not included in these analyses. The tests of heterogeneity were non‐significant for positive/negative affect, *Q* (1) = 0.08, *p* = .78; *I* ^*2*^ = 49.69%, indicating that the effects of gratitude and health behaviours garnered from the 13 studies that tested the partial correlations with future time orientation (*r*_*avg*_ = .275, 95% CI [.24, .31]) were not significantly different from those that were not included in this analysis (*r*_*avg*_ = .264, 95% CI [.20, .33]). Similar results were obtained for positive and negative affect, with the 8 samples that did (*r*_*avg*_ = .265, 95% CI [.22, .31]) and the 16 samples that did not include a measure of affect (*r*_*avg*_ = .275, 95% CI [.23, .32]), having effects that were comparable in magnitude, *Q* (1) = 0.09, *p* = .77; *I* ^*2*^ = 49.69%.

### Moderator analyses – Unadjusted effects of gratitude and health Behaviours

The methodological moderator analysis for sample type was non‐significant, *Q* (2) = 4.52, *p* = .104. The effects garnered from community samples (*r*_*avg*_ = .290; *k* = 14; 95% CI [.25, .33]) were not significantly different from those garnered from the medical (*r*_*avg*_ = .223; *k* = 4; 95% CI [.14, .31]) and student samples (*r*_*avg*_ = .252; *k* = 6; 95% CI [.18, .32]).

The conceptual moderator analysis was also non‐significant, *Q* (1) = 0.12, *p* = .734. The effects garnered from samples which measured gratitude as a trait (*r*_*avg*_ = .271; *k* = 20; 95% CI [.24, .31]) were not significantly different from those garnered from those that measured gratitude as a state (*r*_*avg*_ = .268; *k* = 4; 95% CI [.16, .37]).

The meta‐regression testing the potential influence of participant age was non‐significant, *b* = −0.00 [.00, −.01], *Q*_*model*_ (1) = 0.06, *p* = .81, *Q*_*residual*_ (21) = 43.83, *p* = .01, as was the meta‐regression for participant sex, *b* = 0.04 [−.21, .29], *Q*_*model*_ (1) = 2.68, *p* = .10, *Q*_*residual*_ (24) = 45.64, *p* = .00. These results indicated that the age and percentage of females in the samples did not have a significant influence on the magnitude of the associations between gratitude and health behaviours.

### Moderator analyses – Adjusted effects of gratitude and health Behaviours for FTO

Because there were 13 studies that included a measure of FTO, we conducted moderation analyses for age and participant sex on the adjusted effects. Parallelling the results for the unadjusted effects, the meta‐regression testing participant age was non‐significant, *b* = −0.00 [−.01, .01], *Q*_*model*_ (1) = 0.07, *p* = .81, *Q*_*residual*_ (11) = 27.93, *p* = .01, as was the meta‐regression for participant sex, *b* = 0.27 [−.02, .55], *Q*_*model*_ (1) = 3.34, *p* = .07, *Q*_*residual*_ (11) = 22.32, *p* = .02.

### Tests of publication bias

This Failsafe *N* exceeded the 5 *k* + 10 studies cutoff (130) recommended by Rosenthal (1979). The Failsafe *N* analysis indicated that an additional 3020 studies with non‐significant results for the association of gratitude and health behaviours would need to be included in the set of studies that were statistically meta‐analysed to reduce the *p* value below .05. This suggested a low probability of publication bias.

## DISCUSSION

The current study aimed to extend current theory and research on the role of gratitude for supporting health behaviours, by testing the relative contributions of positive and negative affect and future time orientation (FTO) in the link between gratitude and health behaviours. Extending previous research which found gratitude was associated with individual health behaviours (Fritz et al., 2019; Hill et al., 2013; Legler et al., 2019; Millstein et al., 2016), the current findings found that gratitude, whether trait or state, was associated with an index capturing a range of health‐promoting behaviours across 24 diverse samples. Importantly, this association did not vary as a function of the form of gratitude (trait vs. state), the sample type (student versus community versus medical) or participant demographic characteristics. Consistent with our hypotheses and the tenets of the Temporal Affective Self‐Regulation Resource (TASRR) model (Sirois, 2015a, 2015b), we also found that the magnitude of the pooled associations between gratitude and health behaviours was attenuated when the contributions of positive affect, negative affect and FTO were accounted for.

These findings have several important implications for theory and research on gratitude and health. Although correlational, the current findings align with those from gratitude interventions (e.g., Boggiss et al., 2020) and suggest that gratitude may be equally beneficial for non‐medical, student and chronic illness populations. Much of the research on gratitude interventions with respect to health behaviours in medical populations is limited to a small set of chronic conditions, including cardiovascular conditions (Celano et al., 2018; Legler et al., 2019; Millstein et al., 2016), and diabetes (Schache et al., 2020). Our findings provide evidence that gratitude may also be beneficial for supporting health behaviours in other chronic conditions, including CFS, as well as in non‐medical populations and university students. Cultivating gratitude via gratitude journals or lists (Cheng et al., 2015; McCullough et al., 2002) could therefore be a low resource approach for facilitating uptake of key health‐promoting behaviours in both medical and non‐medical populations.

Although based on correlational data, the findings from this research when viewed from the lens of the TASRR framework (Sirois, 2015a, 2015b) provide plausible explanations for why gratitude may support the practice of health‐promoting behaviours that go beyond previous accounts that focused primarily on positive affect (Schache et al., 2019). From a self‐regulation resource perspective, gratitude provides both affective (high positive affect, low negative affect) and temporal resources that are key for supporting self‐regulation, an essential process for health behaviour change (Sirois & Wood, 2025). Previous research has found that reductions in negative affect explained, in part, why gratitude interventions were effective for increasing healthy eating (Fritz et al., 2019) and sleep quality and duration (Wood et al., 2009). The current study is the first to test the roles of positive affect and FTO alongside low negative affect in the link between gratitude and health behaviours. These findings therefore provide important theoretically grounded insights consistent with self‐regulation theory and the TASRR model (Sirois, 2015a, 2015b).

Examining gratitude in relation to a range of health‐promoting behaviours is noteworthy for several reasons. Previous research has found that gratitude was associated with individual health behaviours, such as eating behaviours or medical adherence. In contrast, the current research extends this evidence by finding that gratitude was associated with an index comprised of different health‐promoting behaviours including healthy eating, activity levels, sleep behaviour and stress management. This evidence also suggests that gratitude may contribute to the self‐regulation of a broader range of health‐promoting behaviours that are motivationally linked. Drawing on the Theory of Triadic Influence (Flay et al., 2009), Lippke et al. (2012) posit that health behaviours from the same domains are more strongly related than those from different domains, in part because their behavioural consequences serve to unify the motivations to engage in these behaviours. Accordingly, the current findings may also extend to other forms of health‐promoting behaviours not assessed in the current study and would therefore be a potentially fruitful area of investigation for future research.

### Limitations and strengths

The findings from this study, though novel, should nonetheless be considered in light of certain limitations and strengths. The moderator subgroup analyses relied upon a small number of studies in each group, indicating that such results should be interpreted with caution. Future research with at least 10–12 studies per subgroup would increase confidence in the robustness of the results found in the current research (Card, 2012). Additionally, not all of the studies included in the overall analysis of gratitude and health behaviours included measures of positive and negative affect, or future time perspective. The findings for these smaller subsets of analyses of the semi‐partial associations should therefore be considered preliminary. There is some evidence that gratitude is viewed and experienced differently between Western and non‐western cultures, especially with respect to the production of positive and negative feelings (Boehm et al., 2011; Titova et al., 2017). As the current study included studies mainly from Western cultures, it is unclear if the current findings would extend to non‐western cultures.

Additionally, it was not possible to test one of the key assumptions of the TASRR model, that positive and negative affect and temporal thinking promote health behaviours through their synergistic, dynamic and mutually reinforcing roles in self‐regulation. Instead, each of the proposed self‐regulation resources was tested separately, due in part to the limited samples that included all three variables. As well, the WBI is a summary measure of health behaviours that did not provide the opportunity to examine how specific behaviours might be more or less strongly related to gratitude. Future research should therefore explore the proposed dynamic processes of the TASRR using daily diary or other longitudinal approaches, and compare the associations of gratitude to different specific health behaviours.

These limitations aside, the current research has several strengths worth noting. Although the MOD analytic approach taken in the current study might be considered somewhat unconventional, it aligns with Cumming (2014) recommendations for approaches that help build a cumulative knowledge base in an under‐studied area. Analysing data sets from the authors' labs also affords the opportunity to control for the contributions of the self‐regulation resource variables suggested by the TASRR model (Sirois, 2015a, 2015b), something that would not have been possible within a traditional meta‐analysis framework given the limited research on gratitude and health behaviours. Estimating the link between gratitude and health behaviours in a traditional meta‐analysis framework would have also meant including a reduced number of studies with individual and varied health behaviours that would introduce a high degree of heterogeneity into the analysis. Similar to other research using a MOD approach (e.g., Sirois, 2020), the use of the same measure of health promoting behaviours in the current analysis reduced this measurement heterogeneity, providing a more precise test of potential moderators.

The meta‐analysis of 24 samples is also a clear strength of the current study. Testing the associations of trait and state gratitude to health behaviours across multiple and diverse samples increased confidence that the results will replicate. This also provides some support for the generalizability of the findings as well as producing a reasonable estimate of the magnitude of the association of gratitude with health‐promoting behaviours. Framing these analyses within the TASRR model (Sirois, 2015a, 2015b) also presented a theoretically plausible explanation for the links between gratitude and health behaviours found in the current study and previous research.

## CONCLUSIONS

Taken together, the findings from the current study provide initial evidence indicating that gratitude is associated with more frequent practice of health‐promoting behaviours, as well as insights into the factors that may contribute to this association. As posited by the TASRR model (Sirois, 2015a, 2015b), we found that the higher levels of positive affect and FTP, and lower levels of negative affect that characterize gratitude, served as self‐regulation resources that accounted for, in part, the association of gratitude with the practice of health‐promoting behaviours. Overall, these findings add to a growing evidence base on the value of gratitude for health outcomes, and alongside previous research demonstrating the effects of gratitude interventions on health behaviours (Boggiss et al., 2020), further highlight gratitude as a potential alternative, strengths‐based approach to health behaviour change across different populations and contexts.

## AUTHOR CONTRIBUTIONS

**Fuschia M. Sirois:** Conceptualization; investigation; writing – original draft; methodology; validation; writing – review and editing; formal analysis; project administration; data curation; resources. **Jameson K. Hirsch:** Writing – review and editing; data curation; resources; methodology; investigation. **Alex M. Wood:** Conceptualization; writing – review and editing; validation.

## Data Availability

The data that support the findings of this study are available from the corresponding author upon reasonable request.
